# Childhood sexual abuse experiences and its associated factors among adolescent female high school students in Arbaminch town, Gammo Goffa zone, Southern Ethiopia: a mixed method study

**DOI:** 10.1186/s12914-015-0059-6

**Published:** 2015-08-18

**Authors:** Aleme Mekuria, Aderajew Nigussie, Muluemebet Abera

**Affiliations:** Department of Public Health Nursing, Arbaminch College of Health Sciences, P.O. Box: 155, Arbaminch, Ethiopia; Department of Population and Family Health, College of Public Health and Medical Sciences, Jimma University, Jimma, Ethiopia

## Abstract

**Background:**

Childhood sexual abuse is a major social problem in Africa including Ethiopia. Moreover, little has been explored about the pattern of childhood sexual abuse in the context of high school students in Ethiopia in general and in Arbaminch town in particular. Thus, the present study aims to assess the prevalence and associated factors of childhood sexual abuse among adolescent female high school students in Arbaminch town.

**Methods:**

A school- based, cross-sectional study was conducted among adolescent female high school students in Arbaminch town from 3^rd^ to 8^th^ March 2014. Both quantitative and qualitative methods of data collection were used. For the quantitative data, a simple random sampling technique was used to select 369 female students from grade ten of the six high schools. A pre-tested, self-administered questionnaire was used to collect the data and then analysis was made using SPSS version 20 statistical packages. For the qualitative component, fourteen in-depth interviews were conducted and analysed based on the thematic areas.

**Result:**

The prevalence of life time rape among adolescent female high school students in Arbaminch town was 11 %. The odds of experiencing life time rape was higher among students who lived alone (AOR = 4.30; 95 % CI: 1.81, 10.24) and among students who lived with their friends (AOR = 3.31; 95 % CI: 1.23, 8.89) than those lived with their parents. The chance of experiencing rape among students who have had no open discussions with their parents about sexuality and reproductive health was higher (AOR = 2.93; 95 % CI: 1.33, 6.45) than those who have had discussions.

**Conclusion:**

This study revealed high levels of childhood sexual abuse among the adolescent female high school students in Arbaminch town. Ever having a discussion about sexuality and reproductive health with parents, living arrangement of the student, and father’s educational status had statistically significant association with childhood sexual abuse. Unwanted pregnancy and abortion were the most common reproductive outcomes of rape. Comprehensive school based reproductive health education, community based awareness creation, open discussions about sexuality and reproductive health matters with students at family level are recommended.

## Background

Researchers use a wide variety of definitions of ‘childhood sexual abuse’. Many general population surveys define it as ‘unwanted sexual contact’ without asking for specific details of the behaviour. However, for the purpose of this study, childhood sexual abuse (CSA) is defined as a self report of unwanted and inappropriate sexual exposure to a child by an older person or penetration in terms of vaginal intercourse. Though a growing body of researchers believe that childhood sexual abuse is dangerously growing worldwide, prevalence rate of cases vary depending on studies done in different contexts [1].

Globally, the sexual victimization of children remains a significant problem. Studies indicate that the risk of CSA is two or more times higher among females than males [2, 3]. Factors like the absence of one or both parents or being raised by stepfather, parental conflicts, family adversity and social isolation have also been linked to a higher risk for CSA. On the other hand, CSA could appear to occur more frequently among underprivileged families because of the disproportionate number of CSA cases reported to child protective services in those coming from lower socioeconomic classes [4].

CSA has a wide number of psychological sequelae. Among these are low self-esteem, anxiety, depression, anger and aggression, post-traumatic stress, dissociation, sexual difficulties, somatic pre-occupation and disorder, self-injurious or self-destructive behaviour, poor school performance, prostitution, delinquency, transmission of abusive behaviour to subsequent generations and most of the various symptoms and behaviours seen in those diagnosed with borderline personality disorder. The long-term consequences of CSA and neglect are not only relegated to the victim, but also impact their families, future relationships, and society. It is a complex societal problem that requires a comprehensive response [5].

In developing countries like Ethiopia, underreporting is common. The problem of obtaining accurate statistics on the prevalence of child and adolescent sexual abuse can be attributed to several factors: inconsistencies in the definitions given to what constitute CSA, fear, social stigma against the rape survivors, and other social and cultural norms are some of the factors. It is committed in “complete secrecy” and most victimised children do not report as they are “too ashamed to talk about it” [6].

A study done on female students in Jimma zone, South West Ethiopia, in 2010, revealed that the prevalence of childhood sexual abuse was 16.6 %, that is experience of sexual assault (*verbal,* such as insulting children using taboo sexual words; *visual*, displaying pornography, forcing one to show his/her sex organ or forcing to see somebody’s sex organ and *physical*, i.e. fondling, touching in a sexual manner, and raping). Moreover, females who were sexually abused had higher mean of self-reported depression scores. They also indicated more incidences of panic, and post traumatic stress disorder (PTSD) syndromes than their counterparts [7].

To the best of our knowledge, most of the studies undertaken in Ethiopia were cross-sectional study designs with only quantitative method of data collection. Qualitative data with regard to estimation of the condition and perpetrators are limited. To fill this gap, the current study involved both qualitative and quantitative methodological approaches of data collection. Moreover, little has been explored about the pattern of CSA in the context of high school students in Southern Ethiopia in general and in Arbaminch town in particular. Thus, this study has been conducted with the aim of assessing the prevalence and identifying predisposing factors of CSA among adolescent female high school students in Gammo Goffa zone, Arbaminch town, Ethiopia.

## Methods

### Study area

The study was conducted from 3^rd^ to 8^th^ March 2014 in Gammo Goffa zone, Arbaminch town. The total population of the town is about 103,965 people [8]. There are three private and three public high schools in the town. The total number of students in all high schools of the town during the time of study was 11,690 of which 4310 were girls.

### Study design

A school-based, cross-sectional study was conducted among randomly selected adolescent female students from each high school in the town by using both quantitative and qualitative methods of data collection.

### Study participants

All randomly selected female students from high schools in the town were the study subjects.

### Inclusion criteria

Regular (day time) grade ten female adolescents who were present in the school on the day of the administration of the questionnaire were included.

### Exclusion criteria

Students who were not able to complete the questionnaire due to serious illness were excluded.

### Sample size and sampling procedure

The sample size for the quantitative component was calculated considering 68 % prevalence of CSA **(5),** 95 % certainty and 5 % of margin of error between population and sample with non response rate 10 %. Therefore, the total calculated sample size for this study was 369 female students.

To supplement the findings of the quantitative study and explore the depth of life time exposure of CSA and its associated factors, fourteen in-depth interviews were conducted. Female school teachers and female students who were not involved in the quantitative study and were thought to be informative (participants of clubs, school mini media) were selected purposively.

**Fig. 1:**
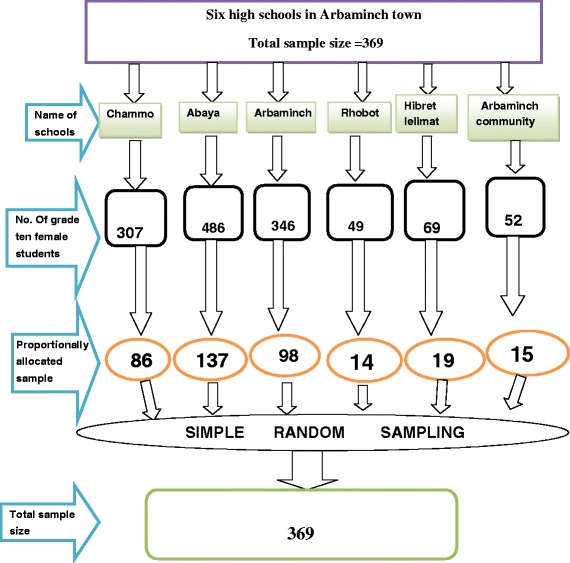
Schematic presentation of sampling procedure among the six high schools in Arbaminch town, March 2014 (*n* = 369)

Place of in-depth interview was selected as to the convenience of the participants. There were six high schools in Arbaminch town. The total sample size was allocated by probability proportional to size (PPS) to the six high schools in the town (Fig. 1).

Grade ten high school students were chosen because the investigators believe that they are matured enough to have the courage to report their sexually abusive experiences. Moreover, their ages are expected not to be too far above the maximum age for adolescents, that is 19 years.

### Data collection

Six female nurses and two health officers were recruited to facilitate the quantitative component of data collection and supervision respectively. Moreover, they were clearly briefed about the purpose of the study and an intensive training on the data collection methods was given for two days.

A pretested, structured, anonymous, self administered questionnaire was used, which was partly adapted from the standard “childhood experience of care and abuse questionnaire (CECAQ)” [9]. The questionnaire was first prepared in English, translated to Amharic then back translated to English language for its consistency by two different individuals who speak both English and Amharic (the local language) fluently.

In order to ensure the quality of the data, pretesting of the questionnaire was done in the same set- up having similar age group but not from a selected high school. Some unclear and difficult questions to understand by most of the students were corrected accordingly during the pre-test. The principal investigator and supervisors supervised the data collection process. The questionnaires were checked for completeness and consistency in daily basis.

In order to maintain confidentiality, the sitting arrangement of the participants was considered; all the selected girls were called and made to sit in prior arranged rooms. Each student took a single seat with sparse arrangement of chairs and desks. No names or identifiers were included on the questionnaire. Students were instructed to fill the questionnaire and leave it in the prepared collecting box. This was followed by an awareness class about the nature and consequences of childhood sexual abuse, preventive measures and coping strategies.

For the qualitative part, verbal consent was obtained from the participants and then the data collectors conducted the interview by using semi-structured interview guide. The interview was entirely tape recorded and field notes were taken.

### Operational definitions and definition of terms

Childhood sexual abuse (CSA)/rape- a self report of unwanted and inappropriate sexual exposure to a female child by an older person or penetration in terms of vaginal intercourse

Extremely poor/below poverty line – a family income < $1.25 USD/per day

Reproductive outcomes**-** if a female student was exposed to unwanted pregnancy, abortion, STIs, HIV/AIDS after experiencing rape

Intra-familiar member- blood related union of the perpetrator to the rape survivor (father, mother, brother, uncle, aunt, nephew, etc…)

Extra- family member- anyone who has no blood related union to the rape survivor (friends, acquaintances, neighbors, etc…)

Verbal harassment *-* insulting children using taboo sexual words, unwanted sexual comments

### Data analysis

For the quantitative component, data were entered in to Epi-data V2.2**,** edited and then exported to SPSS version 20 statistical packages for analysis then cleaned for inconsistencies and missing values. Descriptive statistics including frequencies, percentages, mean, and standard deviations were used to describe findings. The presence of association was assessed using bivariate analysis and associations with a *p*-value <0.05 considered as statistically significant. Multivariate logistic regression was used to control confounding effects and the strength of association was estimated in odds ratio and its 95 % confidence interval. Variables with *p*-value <0.2 in the bivariate analysis were candidates for the final model.

For the qualitative data, the tape recorded interview was thoroughly listened to and fully transcribed to the language of the discussion (Amharic). The final transcribed audio recorded data and field notes were translated into English. Responses and comments were grouped and categorized according to the themes then major findings were narrated and summarized based on thematic areas and finally, writing up and descriptions were performed.

### Ethical consideration

Ethical clearance was obtained from Jimma University Ethical Review Committee. A formal letter was submitted to the Educational office of the Gammo Goffa zone and subsequently to high schools of the Arbaminch town where the study took place. Written permission from the parents of the respondents was obtained a day before the time of data collection. Oral and written permissions from the schools and the respective study subjects were obtained. The study was explained to the subjects and their consent to participate in the study was assured before completing the questionnaire.

## Results

### Socio-demographic characteristics

Out of the expected 369 participants, a total of 362 female students appropriately completed the questionnaire. Seven respondents were excluded due to grossly incomplete and inconsistent responses. Therefore, the study was conducted among 362 female respondents yielding 98 % response rate. Two hundred and twenty one (61 %) of the respondents were between the age group of 16 and 17 years with the mean age of 16.6 ± 1.1 SD years.

Regarding ethnicity, 275(52.7 %) of the respondents were Gammo followed by Amhara 37(10.2 %). Orthodox Christianity was the most frequent religion 185(51.1 %) followed by protestant 147(40.7 %). Three hundred and thirty one (91.4 %) of the students were single. The living arrangement of the students indicated that 196(54.1 %) were living with their parents, 72(19.9 %) with their friends, and 94(26.0 %) alone. One hundred and sixty six (45.9 %) of the students reported that their fathers’ educational status was secondary school complete and above. One hundred and twenty two (34.5 %) respondents came from a family having less than five members (Table 1).

**Table 1:** Socio-demographic characteristics of adolescent female high school students, Arbaminch town, March 2014 (*n* = 362)

Variables		No.	%
Age	14–15	55	15.2
	16–17	221	61.0
	18–19	86	23.8
Religion	Orthodox	185	51.1
	Muslim	8	2.2
	Protestant	147	40.7
	Catholic	11	3.0
	Other	11	3.0
Marital status of the student	Single	331	91.4
	Married	31	8.6
Living arrangement of the student	Father and mother	196	54.1
	Friends	72	19.9
	Alone	94	26.0
Educational status of the student’s father	Below secondary	196	54.1
	Above secondary	166	45.9
Educational status of the student’s mother	Below secondary	254	70.6
	Above secondary	108	29.4
Family size	<5	122	34.5
	5–9	209	59.0
	> = 10	31	6.5

### Reported sexual history

From the total 362 respondents, 79(21.8 %) reported that they were sexually active. The mean age at first sexual debut was 15.12 with ± 1.75 SD years. Twenty seven (34.1 %) of them started sexual intercourse based on their willingness. One hundred and ninety three (53.3 %) of the respondents reported that they knew a child who was raped. Out of the 362 female students, 187(51.7 %) have had open discussions with their parents about sexuality and reproductive health matters (Table 2).

**Table 2:** Reported sexual history among adolescent female high school students in Arbaminch town, March 2014

Variables		No.	%
History of sexual intercourse	Yes	79	21.8
	No	283	78.2
Age at which sexual intercourse started	9–12	8	10.3
	13–16	52	66.7
	17–19	19	23
Reason for starting sexual intercourse	Based on willingness	27	34.1
	Peer pressure	21	26.6
	Engagement in marriage	13	16.5
	Forcefully	9	11.4
	To get money	9	11.4
Know a child who was raped	Yes	193	53.3
	No	169	46.7
Recommendation to alleviate the problem	Stiff punishment of the abuser	126	34.8
	Health announcement in mass media	113	31.2
	School health education	68	18.8
	Reporting to legal body	55	15.2
Ever having a discussion about sexuality with parents	Yes	187	51.7
	No	175	48.3

### Prevalence of childhood sexual abuse

**Fig. 2:**
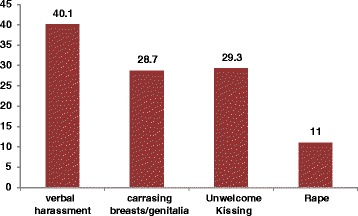
Lifetime prevalence of childhood sexual abuse among adolescent female high school students, Arbaminch town, March 2014

Among the total respondents, 11 % (95 % CI: 8.3, 13.7) reported that they had experienced forceful sexual intercourse (rape) in their lifetime. Caressing breasts or genitals was reported by 28.7 % (95 % CI: 32.5, 39.3) of the students. Similarly, 29.3 % (95 % CI: 24.1, 27.9) reported that they experienced unwelcome kissing (Fig. 2).

**Fig. 3:**
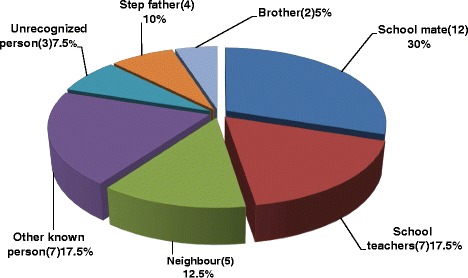
The relationship of the perpetrator to the rape survivor of adolescent female high school students, Arbaminch town, March 2014 (*n* = 40)

Out of the 40 rape survivors, 34(85 %) were raped by extra-familial members and the remaining six (15 %) were raped by family members (Fig. 3). All the rape survivors were raped by male perpetrators. Among the 40 female students who reported to have experienced rape, 12(30 %) responded that the estimated age of the perpetrator was 10 years more as compared to their age. Thirty two (80 %) of those rape survivors were between the age group of 13 and 16 years. Almost half, 20(50 %) of them were living alone during the occurrence of the event (Table 3).

**Table 3:** Perpetrator and condition during rape among adolescent female high school students, Arbaminch town, March 2014 (*n* = 40)

Variables		No.	%
Age of the student at rape	9–12	3	7.5
	13–16	32	80.0
	17–19	5	12.5
Estimated age of the perpetrator	More than 10 years	12	30
	5–10 years	8	20
	1–5 years	4	10
	Equal to my age	5	12.5
	Other	11	27.5
Living with, when the rape occurred	With my parents	10	25
	With my friends	10	25
	Alone	20	50
Place where the rape took place	In the victims house	7	17.5
	In the perpetrators house	19	47.5
	at school	2	5.0
	Hotel	8	20.0
	Other	4	10

From the total 40 rape survivors, 15(37.5 %) of them disclosed their case to someone else. The remaining, 25(62.5 %) didn’t report to anyone else. Among those who disclosed their case, only, seven (46.7 %) reported to legal bodies (police and court), five (33.3 %) to their friends, two (13.3 %) to their parents. The dominant reason for not reporting was fear of their families by 12 (48 %) of the rape survivors. Other reasons stated were fear of stigma, didn’t know what to do, fear of the perpetrator and other unspecified reasons by six (24 %), four (16 %), two (8 %) and one (4 %) respectively. Regarding the action taken against the perpetrators, imprisonment was taken as a legal action only on five (33.4 %) of the perpetrators (not shown in table).

Almost all the study participants suggested possible solutions to combat the problem; of these 126(34.8 %) suggested stiff punishment of the perpetrator, 113(31.2 %) implementing health announcements in the mass media, 68(18.8 %) suggested school health education and 55(15.2 %) recommended reporting to legal bodies.

The qualitative results are in line with the above findings. Most of the key-informants explained that students they knew were raped by their friends and someone very close to the family member. Moreover, sexual abuse by family members was not uncommon: step fathers, even sometimes biological fathers and brothers were involved.

One of the female school directors stated,

*“…..I remember in my village, following the divorce of her parents, a girl was living with her father. The drunkard father came to her bed in the mid night, muffled her mouth with his hands and raped her. The condition happened up on her frequently through time. She came to me and shared her secret including her current pregnancy from her birthfather. I convinced her to report to legal bodies, then her father had been accused of and presented to court, but finally, she couldn’t tolerate the psychological impact. Hence, she disappeared from the town, I haven’t seen her then……”*

**Fig. 4:**
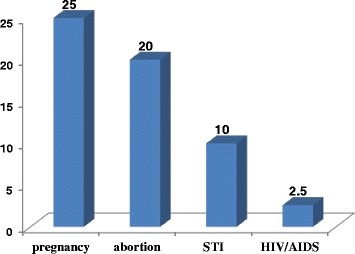
Reported outcomes of childhood sexual abuse among female rape survivors, Arbaminch town, March 2014 (*n* = 40)

From the total 40 girls who reported to have experienced rape, 10(25 %) ended up with an unwanted pregnancy. Of those ten rape survivors who reported to have had an unwanted pregnancy, eight aborted and the remaining two gave live birth. Four of them reported to have genital discharge/lesion and one acquired HIV/AIDS (Fig. 4). The qualitative results also supported this finding: most of the key-informants stated that a number of students that they knew had faced some sort of complications after being raped; unwanted pregnancy and abortion were the common ones they remember.

An 18 year old female student stated that:

*“Students that I know who experienced rape, keep the condition secret. For fear of stigma, they become shameful to buy emergency contraceptives. Therefore, they remain silent then pregnancy follows. Subsequently, they try to terminate it. Due to financial problem, some students go to traditionally working people; the poor hygienic procedure and the trauma lead them in serious illness even some are dying at home.”*

### Factors associated with life time rape

Factors associated with life time rape were assessed. During the bivariate analysis, living arrangement of the student, ever having a discussion about sexuality and reproductive health with parents, father’s educational status, and monthly income had statistically significant association with life time rape. In the bivariate analysis, variables which had statistically significant association and variables which had borderline statistically significant association with the lifetime rape were included in the final model to see the effect of individual variables on the dependent variable while controlling for potentially confounding variables.

In multivariate logistic regression, living arrangement, father’s educational status, ever having a discussion with parents or guardians on sexuality and reproductive health, and monthly income had statistically significant association with CSA (Table 4).

**Table 4:** Multivariate logistic regression analysis results showing association between lifetime rape and selected variables among adolescent female high school students, Arbaminch town, March 2014 (*n* = 40)

Variables	Life time rape		COR(95 % CI)	AOR(95 % CI)
	Yes	No		
Ever having a discussion with parents				
No	29(16.6 %)	146(83.4 %)	*3.17(1.53,6.58)	*2.93(1.33,6.45)
Yes	11(5.9 %)	176(94.1 %	1.00	1.00
Father’s educational status				
Below secondary	34(17.3 %)	162(82.7 %)	*5.59(2.28,13.9)	*4.69(1.84,11.95)
Above secondary	6(3.6 %)	160(96.4)	1.00	1.00
Living arrangement				
Father & mother	10(5.1 %)	186(94.9 %)	1.00	1. 00
Friends	10(13.9 %)	62(86.1 %)	*3.00(1.19, 7.54)	*3.31(1.23, 8.89)
Alone	20(21.3 %)	74(78.7)	*5.02(2.24,11.24)	*4.30(1.81, 10.24)
Monthly income				
<=712.5 ETB (37.5 USD)	29(20.9 %)	110(79.1 %)	* 5.08(2.44, 10.55)	* 3.82(1.76, 8.31)
>712.5 ETB (37.5 USD)	11(4.9 %)	212(95.1 %)	1.00	1.00

*statistically significant at *P* < .05

The odds of experiencing life time rape among students who were living with their friends was higher than those who were living with their parents. (AOR = 3.31; 95 % CI: 1.23, 8.89).

The odds of experiencing life time rape was higher among students who lived alone than those who lived with their parents (AOR = 4.30; 95 % CI: 1.81, 10.24). Students who did not have an open discussion about sexuality and reproductive health with their parents were about three times more likely to experience rape as compared to those students who have had an open discussion with their parents (AOR = 2.93; 95 % CI: 1.33,6.45). The odds of experiencing rape among students who had family income < =712.5ETB (37.5 USD)/month was about three times higher than those students with monthly income > 712.5 ETB (37.5 USD/month (AOR = 3.82; 95 % CI: 1.76, 8.31).

## Discussion

To the best of the authors’ knowledge, this is the first school-based study in Arbaminch estimating the prevalence of CSA and factors associated with it. From the total seven governmental and non-governmental high schools in the town, six high schools participated and 369 sampled female students were included in the study.

The lifetime prevalence of rape among adolescent female high school students in Arbaminch town was 11.0 %. This finding is consistent with studies conducted among school adolescents in: Wolayta Sodo, Ethiopia (8.7 %), Tanzania (8.7 %), Debark, North West Ethiopia (8.8 %), and Addis Ababa, Ethiopia (12.7 %), [10–13].

This finding is much lower than a study conducted among teenagers in South West Nigeria, which was 42.1 % [14]. This discrepancy may be due to social and cultural variation between the study subjects in reporting CSA; the chance of engaging in marriage is considered as minimal for the female rape survivor in Ethiopia. Therefore, they keep it secret for fear of the stigma and other negative responses from the community. From those points of view, under reporting might be there in our case.

The common perpetrators in our study were school mates/friends (30 %) followed by school teachers (17.5 %) and neighbours (12.5 %). From family members, step fathers and brothers were the leading perpetrators. This finding is consistent with a study conducted among school children in Addis Ababa and Jimma, which showed that more episodes of CSA were perpetrated by someone they closely know. The most frequent perpetrators were intimate partners, family members and teachers [11].

Similarly, this finding is consistent with a study conducted in Bahir-dar and Tanzania, Dareselam school children, which showed that the most common perpetrators were neighbours, teachers, peers, family members and friends [12, 15]. This was also supported by the qualitative findings where most of the key-informants explained that students they knew were abused by their friends or someone who frequently visits their family. Fathers and brothers were also involved from family members.

Students who haven’t had open discussions about sexuality and reproductive health with their parents were at higher risk of experiencing CSA as compared to those students who have had open discussions. This finding is consistent with a study done in Bahir-dar town where the odds of experiencing lifetime rape was much higher among students who never had open discussions than those who have had it [15]. This might be because open discussions with parents or guardians about sexuality and reproductive health matters is considered a shame and taboo in our culture. Henceforth, most of the rape survivors were lacking this behaviour. Consequently, this leads to missing opportunities of getting experiences and life skills from parents against the prevention of CSA and escaping mechanisms when conditions arise.

In this study, those female students living alone and those living with their friends were at higher risk of experiencing CSA than those who were living with their parents. This finding is consistent with a study done among high school students in Harar, Eastern Ethiopia; it showed that living with parents had a protective effect on experiencing sexual abuse as compared to living alone [16]. The finding is also comparable with a study done in South East Nigeria that showed childhood sexual abuse was commoner among students who did not live with their biological parents than those lived with their biological parents [14]. The possible explanation could be, children who live with their parents are under direct monitoring and follow up that minimize their chance of exposure to opportunistic predators. Moreover, the parents will care more; cater for their growth and development more than friends and relatives.

Father’s educational status was significantly associated with CSA in that it had preventive effect on experiencing CSA. This finding is in line with a study conducted in Ambo University, where achieving secondary education by the father was associated with decreased sexual abuse [17].

However, the finding was inconsistent with other studies [15, 16]. In our study, the possible reason could be as fathers’ educational level is higher, they may give more attention to their daughters, understand their needs, discuss reproductive and sexual issues freely thereby transferring life skills could be easy; this strengthens the decision making capacity of their daughter against sexually abusive advances.

Monthly income was significantly associated with CSA. The likelihood of experiencing CSA among students who had family income < =712.5ETB (37.5 USD)/month was just higher than those students with monthly income > 712.5 ETB (37.5USD)/month. This finding is in line with a study conducted in Jimma University Specialized Hospital adolescent female students that all raped female survivors belonged to low income categories [18]. The possible explanation could be the economical dependence of female students necessitates them to seek financial support and gifts from males. This deception negatively affects their decision making in their sexual sphere thereby leading them to easy exposure to sexual abuse.

An unwanted pregnancy, abortion, genital discharge/lesion and acquiring HIV/AIDS were the consequences of rape reported by the rape survivors. This finding is consistent with a study done in Harar and Jimma high school students which showed that unwanted pregnancy, abortion, and STIs were the most common consequences of rape [16, 19]. This result is also supported by the qualitative findings that most of the key-informants stated that a number of students that they knew had faced some sort of complications after being raped; unwanted pregnancy and abortion were the common ones.

The reported outcome of HIV and STI is much lower than a study done in Jimma University Specialized Hospital [18]. This difference could be due to methodological variations; in the case of the Jimma study, the result was confirmed based on the test result of HIV and STIs, where as in the present case, it was merely self report. Moreover, our respondents might have not been checked for their sero- status of HIV after being raped. Hence forth, under reporting could be there.

### Study limitations

This study is limited by its cross-section nature, whereby it may not explain the temporal relationship between the outcome variable and some explanatory variables; this limits interpretation of the estimated associations. From the very nature of the study, it assesses personal and sensitive issues related to sexuality which might have caused underreporting experiences of sexual abuse. Thus, the findings of this study should be interpreted within these limitations.

## Conclusion

This study revealed high levels of CSA in Arbaminch town. Living arrangement, ever having a discussion about sexuality and reproductive health with parents, father’s educational status and monthly income were significantly associated with CSA. Discussion with parents or guardians about sexuality and reproductive health has a preventive effect on CSA. Students who were living alone or with their friends were at higher risk of experiencing CSA than those living with their biological parents. Students who reported to have experienced sexual abuse ended up with different negative reproductive outcomes: unwanted pregnancy and abortion were the most common ones.

We recommend that parents should monitor and give due attention to female school children. As far as possible, female children should attend their school living with their family or responsible caretaker rather than left alone or with their friends. The importance of open discussions about sexuality and reproductive health with female school children is of paramount. Therefore, parents have to break their silence against CSA and discuss all about the nature and its negative consequences. Policy makers are urged to work on provision of comprehensive school based reproductive health education including victimization prevention programs and life skill training must be provided besides formal education of female children.
